# Protocol for the generation and assessment of functional macrophages from mouse bone marrow cells

**DOI:** 10.1016/j.xpro.2025.103706

**Published:** 2025-03-20

**Authors:** Andrea Scafidi, Frida Lind-Holm Mogensen, Alessandro Michelucci

**Affiliations:** 1 Neuro-Immunology Group, Department of Cancer Research, Luxembourg Institute of Health, 1210 Luxembourg, Luxembourg; 2 Faculty of Science, Technology and Medicine, University of Luxembourg, 4365 Esch-sur-Alzette, Luxembourg

**Keywords:** Cell biology, Cell culture, Cell isolation, Immunology, Model organisms, Molecular biology

## Abstract

Macrophages play essential roles in tissues, wherein they exert beneficial or detrimental functions depending on the signals they encounter during their differentiation. Here, we present a protocol to differentiate mouse bone marrow cells into macrophages under specific cues to evaluate their impact on macrophage phenotypic acquisition. We provide detailed instructions for optimal cell culture conditions, quality controls, and examples of concluding immunological functional assays. This protocol is applicable in short- and long-term drug-based modulation of macrophage functions.

For complete details on the use and execution of this protocol, please refer to Scafidi et al.^1^

## Highlights

• Protocol to differentiate mouse bone marrow-derived macrophages under precise cues
• Combines optimal cell culture conditions and quality control analyses
• Allows analysis of resultant macrophage immunological functions
• Enables evaluation of drug effects on macrophage phenotypic acquisition

## Abstract (editor highlights)

Publisher’s note: Undertaking any experimental protocol requires adherence to local institutional guidelines for laboratory safety and ethics.

## Graphical abstract

**Figure:**
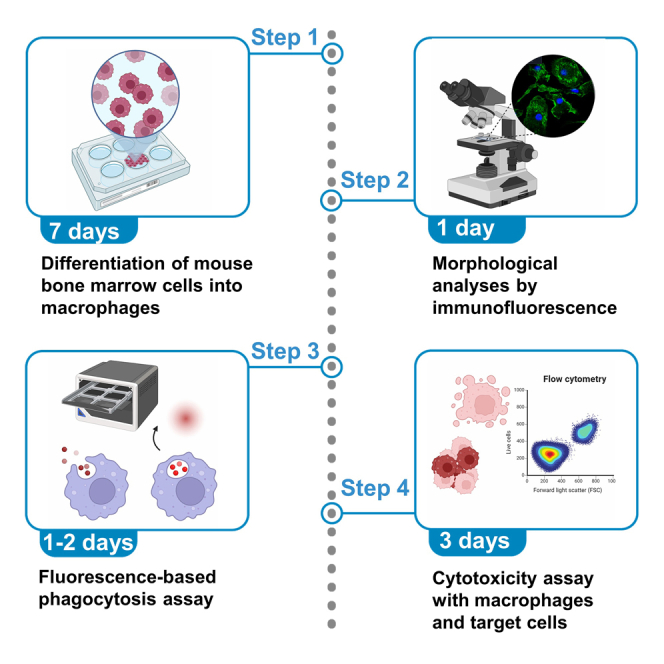

## Before you begin

Macrophages are myeloid cells that play critical roles in maintaining homeostatic conditions. Tissue-resident macrophages derive from embryonic and fetal progenitors persisting in the adult tissues based on their self-renewal capacity. Under inflammatory conditions, such as infections or tumorigenic processes, bone marrow-derived macrophages invade the inflamed tissue to restore homeostasis. These cells derive from bone marrow hematopoietic stem cells that mature into myeloid progenitors and migrate into the blood stream as monocytes.^2^ Once in the blood, monocytes are recruited to the tissue via cytokine signaling, such as CCL2-CCR2 interaction, where they differentiate into macrophages.^3^ Under threatening conditions, macrophages can display either beneficial or detrimental features, thus actively participating in the resolution of the compromising environment or contributing to the development and progression of disease settings. Due to their critical roles, therapies aimed at skewing macrophages towards a desired phenotype are on the rise. However, while common *in vitro* models serve to assess the effect of a treatment on fully differentiated macrophages, the influence of specific cues during their differentiation from bone marrow cells is less investigated. Therefore, we here define a detailed protocol to assess the effects of particular cues on mouse macrophage phenotypic acquisition. In addition to the description of optimal cell culture conditions, we define quality controls to assess the macrophage phenotype by multicolor flow cytometry analyses and provide systematic instructions to verify changes of key immunological features, including phagocytosis and cytotoxicity.

### Institutional permissions (if applicable)

This protocol requires the usage of mice. Therefore, before starting the following procedures, check the guidelines provided by the animal welfare committee and the national authorities to conduct animal experimentation and obtain the relevant authorizations. The experiments performed in this protocol were not subject to a project approval due to the severity of the procedures set as “non-recovery” according to the Luxembourgish legislation about animal experimentation (Règlement grand-ducal adopted on January 11th, 2013). However, these procedures were performed in accordance with rules and guidelines established by the animal welfare committee of the University of Luxembourg.

## Key resources table

**Table:** 

REAGENT or RESOURCE	SOURCE	IDENTIFIER
**Antibodies**		

TruStain FcX (0.5 μL/tube)	BioLegend	Cat# 101320; RRID: AB_1574975
PerCP_Cy5.5 anti-CD11b (2.5 μL/tube)	BioLegend	Cat# 101227; RRID: AB_893233
PE anti-F4/80 (2.5 μL/tube)	BioLegend	Cat#123110; RRID: AB_893486
BV510 anti-CD80 (1.25 μL/tube)	BioLegend	Cat# 104741; RRID: AB_2810337
BV605 anti-CD86 (2.5 μL/tube)	BioLegend	Cat# 105037; RRID: AB_11204429
PE/Cy7 anti-H2Kb/H2Db (1.25 μL/tube)	BioLegend	Cat# 114615; RRID: AB_2750195
APC anti-I-Ab (0.25 μL/tube)	BioLegend	Cat# 116418; RRID: AB_10574160

**Chemicals, peptides, and recombinant proteins**		

Paraformaldehyde, 4% in PBS	Fisher	Cat# 15424389; CAS: 30525-89-4
Ethanol 70% DAB	Carl Roth	Cat# 7301.1; CAS: 64-17-5
Ammonium chloride	Sigma	Cat# A9434; CAS: 12125-02-9
Acti-Stain 488 fluorescent phalloidin (1:300)	Cytoskeleton, Inc.	Cat#PHDG1
ROTI Cell 10× PBS	Carl Roth	Cat# 9150.1
Trypan blue	Fisher (Gibco)	Cat# 11538886; CAS: 72-57-1
DMEM, high glucose, GlutaMAX supplement	Fisher	Cat# 61965026
Sodium pyruvate (100 mM)	Fisher (Gibco)	Cat# 12539059; CAS: 113-24-6
Penicillin-streptomycin (10,000 U/mL)	Fisher (Gibco)	Cat# 11548876; CAS: 8025-06-7
HEPES (1M)	Fisher	Cat# 11560496; CAS: 7365-45-9
Recombinant mouse M-CSF protein, 10 μg	Bio-Techne (R&D Systems)	Cat# 416-ML-010/CF
Fetal bovine serum, qualified, Brazil	Fisher (Gibco)	Cat# 10270-106
Metformin hydrochloride	Merck	Cat# PHR1084; CAS: 1115-70-4

**Critical commercial assays**		

LIVE/DEAD Fixable Near-IR	Invitrogen	Cat# L34975
CellTrace CFSE Cell Proliferation Kit	Invitrogen	Cat# 15598431
TO-PRO-3 Iodide (642/661), 1 mM solution in dimethyl sulfoxide (DMSO)	Fisher	Cat# T3605; CAS (DMSO): 67-68-5
pHrodo BioParticles Conjugates for Phagocytosis and Phagocytosis Kit, for Flow Cytometry	Invitrogen	Cat# P35361
BD CompBeads Anti-Mouse Ig, κ/Negative Control Compensation Particles Set	BD	Cat# 552843; RRID: AB_10051478
BD CompBeads Anti-Rat and Anti-Hamster Ig κ /Negative Control Compensation Particles Set	BD	Cat# 552845; RRID: AB_10058522
ArC Amine Reactive Compensation Bead Kit	Invitrogen	Cat# A10628

**Experimental models: Cell lines**		

GL261	DSMZ	ACC-802; RRID:CVCL_Y003

**Experimental models: Organisms/strains**		

C57BL/6NCrl, 2–6 months of age	Charles River	RRID: IMSR_CRL:027

**Software and algorithms**		

CellProfiler Image Analysis Software	Broad Institute	Stirling et al.^4^; RRID: SCR_007358
NovoExpress Software 1.6.2	Agilent	RRID: SCR_024676
FlowJo 10.10	BD	RRID: SCR_008520

**Other**		

Disposable syringe Omnifix With Luer-Lock fitting, 20 mL	Carl Roth	Cat# T550.1
BD Microlance Hypodermic Needles 26G × 3/8″	BD	Cat# 300300
Cell scraper	Greiner	Cat# 541070
Nunc non-treated multidishes; 6-well plates	Fisher	Cat# 150239
FACS tubes (5 mL polystyrene round-bottom tube)	Falcon	Cat# 352052
NovoCyte Quanteon Flow Cytometer Systems 4 Lasers	Agilent	RRID:SCR_025831
Sartorius IncuCyte S3 Live Cell Analysis System	Sartorius	RRID:SCR_023147
Nikon Eclipse TE2000 inverted microscope system	Nikon	RRID:SCR_023161
Coverslips 12 mm, 0.13–0.16 mm	Epredia	Cat# CB00120RA120MNZ0
Glass slides 76 × 26 mm	Roth	Cat#L192
Tweezer	Brand of choice	

## Materials and equipment

### Recombinant mouse M-CSF protein

• Prepare recombinant mouse M-CSF protein by dissolving 10 μg of M-CSF powder in 1 mL PBS supplemented with 0.1% bovine serum albumin (BSA) to obtain a final solution of 10 μg/mL. Store at −20°C for maximum one month or at −80°C for maximum three months.

**CRITICAL:** Do not freeze and thaw several times. Do not dilute more than a final concentration of 10 μg/mL for −20°C storage.

### PBS 1×

• Prepare 1 L of 1× PBS by adding 100 mL of 10× PBS to 900 mL of Milli-Q water.
• Filter with 0.22 μm filters for sterilization.
• Store at 4°C for maximum six months.

### Complete DMEM

**Table:** Prepare the complete DMEM according to the following recipe

Component	Concentration	Final volume
DMEM, high glucose, GlutaMAX Supplement	N/A	435 mL
Fetal bovine serum	10%	50 mL
Penicillin/Streptomycin	1%	5 mL
Sodium pyruvate	1%	5 mL
HEPES	1%	5 mL

Store at 4°C for maximum three months.

### Ammonium chloride stock solution

Dissolve 9.09 g of ammonium chloride (NH_4_Cl) in 100 mL of sterile Milli-Q water to obtain a solution of 1.7M. Store at 21°C for maximum six months.

### Drug solution

Prepare the compound of interest in a suitable solvent depending on the drug. For toxic compounds, perform the preparation under a chemical hood. In case of usage of toxic solvents (i.e., dimethyl sulfoxide), take into account relative control cells treated with the same volume of solvent. For treatment of bone marrow cells with metformin, prepare stock solution diluting 500 mg of metformin hydrochloride (powder) in 302 mL of Milli-Q water to obtain a final concentration of 10 mM. Store 10 mM metformin solution at 4°C for maximum 3 months.

### Bovine serum albumin stock solution

Mix 1 g of BSA in 10 mL of 1× PBS to obtain a stock solution of 10% BSA in PBS. Keep the solution on a tube shaker until complete dissolution of the BSA powder. Store BSA stock solution at −20°C for maximum one year.

### Antibody mix

Prepare the antibody mix according to the following recipe. Multiply the amount of each component by the number of samples to be stained plus an additional one to take into account pipetting error. Prepare the antibody mix the day of the usage. Include controls, such as Live/Dead Near-IR only and fluorescence minus one controls (FMOs).

**Table:** Antibody mix

Antibody	μL/tube
APC anti-I-Ab	0.25
PE/Cy7 anti-H2Kb/H2Db	1.25
PerCP_Cy5.5 anti-CD11b	2.5
PE anti-F4/80	2.5
Live/Dead Near-IR	0.5
BV510 anti-CD80	1.25
BV605 anti-CD86	2.5
Brilliant Stain buffer	14.25

## Step-by-step method details

### Bone marrow cell extraction and treatment

**Timing: 7 days**

**Figure 1:**
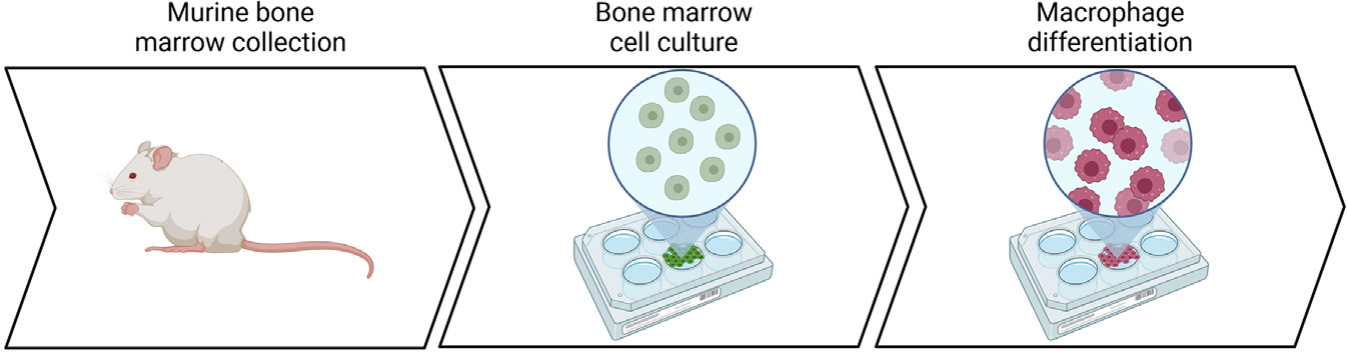
Schematic representation of the protocol used to differentiate mouse bone marrow cells into macrophages

The following section describes the steps to generate bone marrow-derived macrophages (BMDMs) and determine the effect of drug treatment on their differentiation (Figure 1).

**Figure 2:**
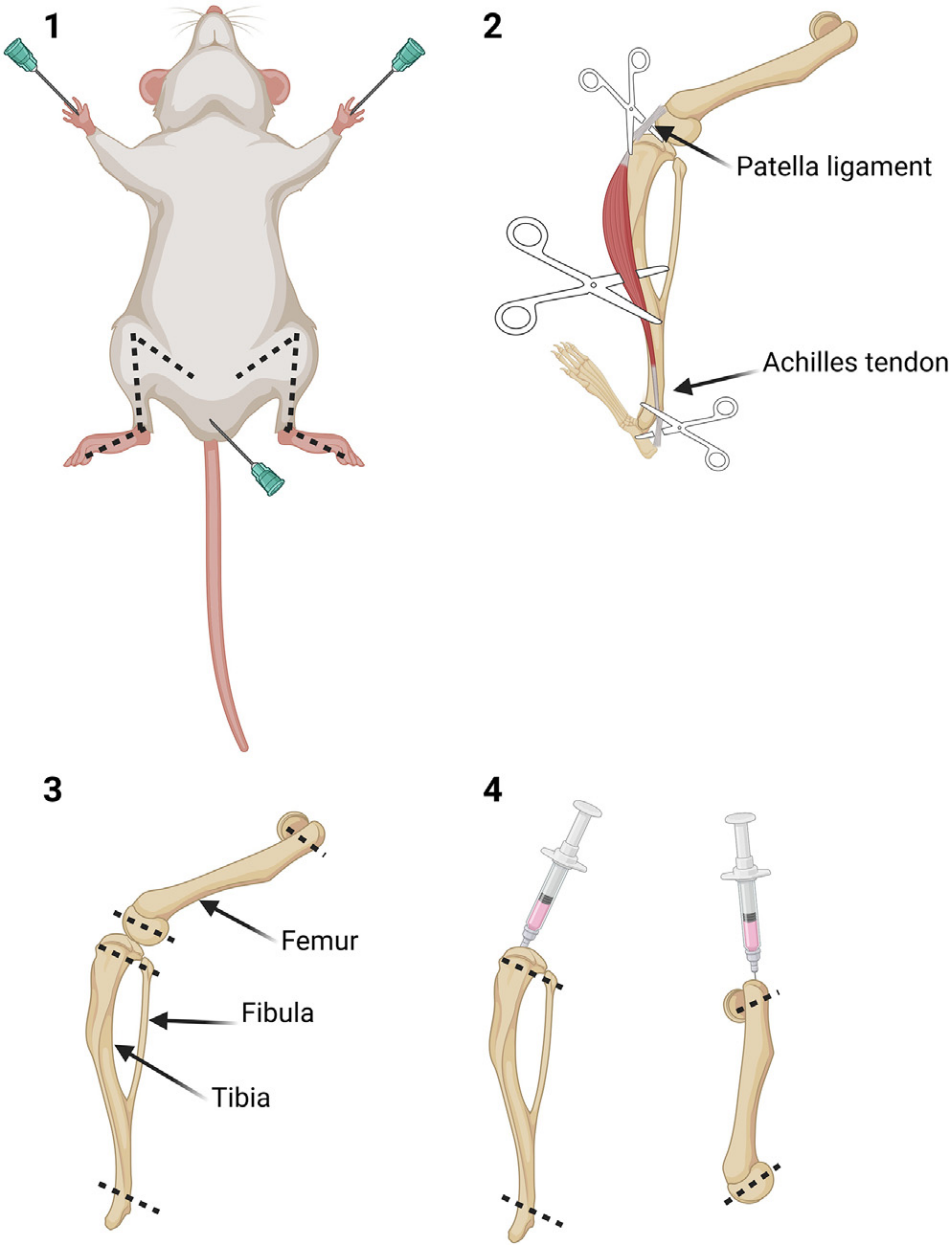
Schematic representation of mouse leg dissection and bone marrow cell extraction

1. Carry out cervical dislocation in accordance with best practices for animal welfare. Preferentially use mice between 2 and 6 months of age to maximize the yield of bone marrow cells.
2. Dissect posterior legs using forceps and scissors according to the following steps (Figure 2):
  a. Attach the mouse in supine position to a stable surface using needles or tape leaving the posterior legs free.
  b. Disinfect the mouse skin and fur using 70% ethanol.
  c. Cut the skin layer using the scissors from the foot pad towards the abdominal cavity.
  d. Detach the skin from the muscular layer using the forceps.
  e. Remove the skin from the foot via pulling it away using the scissors.
  f. Cut the majority of muscle and fat that surround the tibia, fibula and femur using the scissors.
  g. Position the scissors on the ilium femur joint and pull the leg until detached. If needed, cut with the scissor the surrounding tissue to facilitate the detachment.
  h. Store the leg at 4°C in a tube filled with 1× PBS.
  i. Perform the same procedure on the second leg.
3. Wash the legs by submersion in tube containing 70% ethanol for 5 s.
4. Wash the legs by submersion in tube containing 1× PBS for 5 s.
5. Keep the legs in 10 cm petri dish with complete DMEM.
6. Collect tibia and femur from each leg following the further steps:
  a. Cut the Achilles tendon and remove the foot using the forceps.
  b. Cut the patella ligament and the surrounding muscle to detach femur from tibia and fibula.
7. Remove the muscle and the fat from the bone using cotton gauze until the bones are completely exposed avoiding breaking the bones.

***Note:*** Fibula is a very thin bone that contains very few bone marrow cells. For this reason, the user can remove the fibula from the tibia breaking it with the gauze.

8. Load a 10 mL syringe with complete DMEM
9. Attach a 26G × 3/8″ needle to the syringe
10. Flush the bones by inserting the needle in the cavity, according to the following instructions:
  a. Cut the extremities of the bone using the scissors or a bone trimmer.
    ***Note:*** Do not cut the whole extremity of the bones to avoid cell loss. One millimeter of bone is enough to get access to the bone cavity.
  b. Insert the needle in the cavity and flush the bone with 5 mL of medium into a 50 mL tube.
  c. Invert the bone and flush the bone again with 5 mL of medium.
  d. Perform the flush with all the bones.
11. Filter the cell suspension using 70 μm filter into a 50 mL tube.
12. Centrifuge the cell suspension for 7 min at 300 × g at 21°C.
13. Prepare NH_4_Cl 0.17 M working solution diluting 200 μL of NH_4_Cl 1.7 M stock solution in 1800 μL of sterile Milli-Q water.
14. Remove the supernatant and add 2 mL of NH_4_Cl 0.17 M to lyse red blood cells.
15. Incubate at 21°C for 5 min.
16. Dilute the cell suspension with 10 mL of 1× PBS.
17. Centrifuge the cell suspension for 7 min at 300 × g at 21°C.
18. Remove the supernatant and add 10 mL of complete DMEM.
19. Dilute 10 μL of cell suspension in 10 μL of trypan blue.
20. Pipette 10 μL of cell suspension and trypan blue into the Neubauer chamber for cell counting.
21. Count trypan blue negative cells at 10× magnification.

***Note:*** The suspension will be composed of big cells and small particles, which are remaining red blood cells. Exclude red blood cells for further processing.

22. Dilute the cells in complete DMEM to obtain a concentration of 5 × 10^6^ cells/mL.
23. Supplement the cell suspension with 40 ng/mL of M-CSF.

***Note:*** To perform phalloidin staining, add sterile uncoated coverslips to the desired well before seeding the cells.

24. Add 1 mL of cell suspension into each well of 6-well plate.
25. Treat bone marrow cells according to the following steps:
  a. Prepare the drug solution concentrated two times more than the desired concentration in complete DMEM. For metformin treatment:
    i. Dilute metformin stock solution 1:10 in complete DMEM to obtain a final concentration of 1 mM.
    ii. Dilute metformin stock solution 1:5 in complete DMEM to obtain a final concentration of 2 mM.
    iii. Dilute metformin stock solution 1:2.5 in complete DMEM to obtain a final concentration of 4 mM.
    iv. Prepare control solution diluting Milli-Q water 1:2.5 in complete DMEM.
  b. Add 1 mL of drug solution into each well to treat bone marrow cells. For metformin treatment:
    i. Add 1 mL of metformin 1 mM in the wells already containing 1 mL of cell suspension to obtain a final metformin concentration of 0.5 mM.
    ii. Add 1 mL of metformin 2 mM in the wells already containing 1 mL of cell suspension to obtain a final metformin concentration of 1 mM.
    iii. Add 1 mL of metformin 4 mM in the wells already containing 1 mL of cell suspension to obtain a final metformin concentration of 2 mM.
    iv. Add 1 mL of control solution in the wells already containing 1 mL of cell suspension.

***Note:*** Use concentrated drugs in order to avoid use of high concentrations of solvent (DMSO, water, PBS depending on the drug).

26. Incubate 6-well plates at 37°C and 5% CO_2_ for 3 days.
27. Replenish the media by adding 1 mL complete DMEM supplemented with 20 ng/mL M-CSF and the compound at the desired concentration. For metformin treatment:
  a. Dilute metformin stock solution 1:20 in complete DMEM to obtain a final concentration of 0.5 mM.
  b. Dilute metformin stock solution 1:10 in complete DMEM to obtain a final concentration of 1 mM.
  c. Dilute metformin stock solution 1:5 in complete DMEM to obtain a final concentration of 2 mM.
28. Incubate 6-well plates at 37°C and 5% CO_2_ for 4 additional days.

### Quality control of bone marrow-derived macrophage differentiation by multicolor flow cytometry

**Timing: 3 h**

This step provides the assessment of fully differentiated BMDMs based on the expression levels of specific cell surface markers at day 7 of differentiation. Additionally, it is possible to include in the flow cytometry panel additional activation markers specific to the scientific question. Specifically, the abovementioned panel includes markers related to macrophage antigen presentation capacity, such as I-Ab (MHC class II), H2Kb/H2Db (MHC class I), and T cell costimulatory molecules, such as CD80 and CD86.

29. To harvest treated and control BMDMs after 7 days of differentiation, remove the supernatant from each well.
30. Prepare PBS supplemented with 0.2% BSA by diluting 1 mL of 10% BSA in PBS stock solution in 49 mL of 1× PBS.
31. Wash cells by gently adding 2 mL/well of PBS supplemented with 0.2% BSA.
32. Remove the supernatant from each well.
33. Gently add 2 mL/well of PBS supplemented with 0.2% BSA.
34. Scrape off the cells using a cell scraper and collect into 15 mL tubes.

***Note:*** Inspect the well after scraping to be sure that all the cells are collected. After specific treatments, for example following stimulation with pro-inflammatory cytokines, macrophages become firmly adherent and difficult to detach. The usage of dissociation reagents, such as trypsin or accutase, can be taken into account. For further information, check Troubleshooting section.

35. Centrifuge the cell suspension for 7 min at 300 × g at 4°C.
36. Remove the supernatant from each tube.
37. Resuspend the cells in 1 mL/tube PBS supplemented with 0.2% BSA.
38. Dilute 10 μL of cell suspension in 10 μL of trypan blue.
39. Add 10 μL of cell suspension and trypan blue in the Neubauer chamber or other supports for cell counting.
40. Count trypan blue negative cells at 10× magnification.
41. Add 5 × 10^5^ cells/tube into 5 mL round-bottom polystyrene tubes, at least one tube per condition.
42. Prepare samples for compensation controls:
  a. Add 5 × 10^5^ cells in one tube and treat with 5% DMSO to induce cell death for 5 min at 37°C. Alternatively, add one droplet of ArC Amine Reactive Compensation Bead and one droplet of negative control.
  b. Add one droplet of BD CompBeads Anti-Rat and Anti-Hamster Ig κ and one droplet of Negative Control Compensation particles in six tubes.
  c. Add one droplet of BD CompBeads Anti-Mouse Ig, κ and one droplet of Negative Control Compensation particles in one tube.
43. Prepare samples for “FMO” and “unstained” controls:
  a. Add 5 × 10^5^ cells in one tube that will be used as unstained negative control.
  b. Add 5 × 10^5^ cells/tube in each tube that will be used as FMO. For this panel, prepare six FMO tubes.

***Note:*** Compensation beads relate to the used antibodies, therefore compensation beads must be aligned with the host of the antibodies. Additional controls can be included: cells stained with only one antibody (single color control) can be used to improve compensation (see Troubleshooting section, Problem 1) and the inclusion of isotype control antibodies can be used to detect non-specific labeling.

44. Wash cells by adding 3 mL/tube PBS supplemented with 0.2% BSA.
45. Centrifuge all the tubes for 7 min at 300 × g at 4°C.
46. Prepare TruStain FcX solution mixing 0.5 μL/sample of TruStain FcX with 24.5 μL/sample of Brilliant Stain buffer.
47. Prepare the LIVE/DEAD Fixable Near-IR according to the following steps:
  a. Resuspend the dye adding 50 μL of DMSO as per manufacturer instructions.
  b. Dilute the dye 1:10 in PBS.

***Note:*** TruStain FcX cell treatment is used to avoid non-specific staining mediated by binding of immunoglobulin constant fragments (fc) and their receptors.

48. Remove the supernatant from each tube.

***Note:*** Carefully remove as much liquid as possible to avoid further dilution of the antibodies. The usage of an aspirating pump is recommended.

49. Perform TruStain FcX treatment:
  a. “FMO” and “unstained” control samples: add 25 μL/tube of TruStain FcX solution to control sample tubes and resuspend the cells.
  b. Analysis samples: add 25 μL/tube of TruStain FcX solution to analysis sample tubes and resuspend the cells.
  c. Compensation samples: add 25 μL/tube of PBS supplemented with 0.2% BSA to the compensation sample tubes and resuspend it.
  d. Incubate all the tubes for 5 min at 4°C.
50. Perform the staining of the different samples.
  a. Compensation samples:
    i. Prepare the single-antibody solution for compensation controls by mixing one antibody or LIVE/DEAD Fixable Near-IR with PBS supplemented with 0.2% BSA up to 25 μL. The volumes of the used antibodies are listed in the antibody mix recipe above.
    ii. Add the single-antibody solution to each compensation sample and resuspend it.
  b. Live/Dead Near IR compensation sample:
    i. Prepare the Live/Dead Near-IR only solution mixing 0.5 μL of Live/Dead Fixable Near-IR with 24.5 μL of PBS supplemented with 0.2% BSA.
    ii. Add Live/Dead Near-IR only solution to the tube and resuspend it.
  c. Unstained control sample:
    i. Add 25 μL of PBS supplemented with 0.2% BSA to the tube and resuspend it.
  d. FMO control samples:
    i. Prepare FMO solutions mixing all but one the antibodies used for the antibody mix. For each FMO solution, omit a different antibody to have FMO controls for each antibody.
    ii. Add FMO solutions to respective tube and resuspend them.
  e. Analysis samples:
    i. Prepare the antibody mix according to the recipe.
    ii. Add 25 μL of antibody mix to each analysis sample tube and resuspend it.

***Note:*** Prepare a master mix solution for all the samples to reduce the variability. The antibody mix recipe may need to be optimized for the available flow cytometer. Similar optimizations have to be performed if the used antibodies are coupled with different fluorochromes than the ones mentioned in this protocol. Due to the use of Brilliant Violet dyes, we used Brilliant Stain buffer. If other non-Brilliant Violet conjugated antibodies are selected, Brilliant Stain buffer can be substituted with PBS supplemented with 0.2% BSA. Inclusion of isotype control antibodies or FMO controls can be useful for optimal determination of positivity threshold.

51. Incubate all the tubes for 30 min at 4°C protected from the light.
52. Wash the cells by adding 3 mL/tube of PBS supplemented with 0.2% BSA.
53. Centrifuge the cell suspension for 7 min at 300 × g at 4°C.
54. Remove the supernatant from each tube.
55. Resuspend the cells in 100 μL/tube.
56. Resuspend the cells by pipetting.
57. Set-up NovoCyte Quanteon flow cytometer using NovoExpress Software.

***Note:*** These settings are based on the fluorochromes used in this protocol and flow cytometer. Readapt the settings according to the available instrument and any changes in antibody mix.

**Figure 3:**
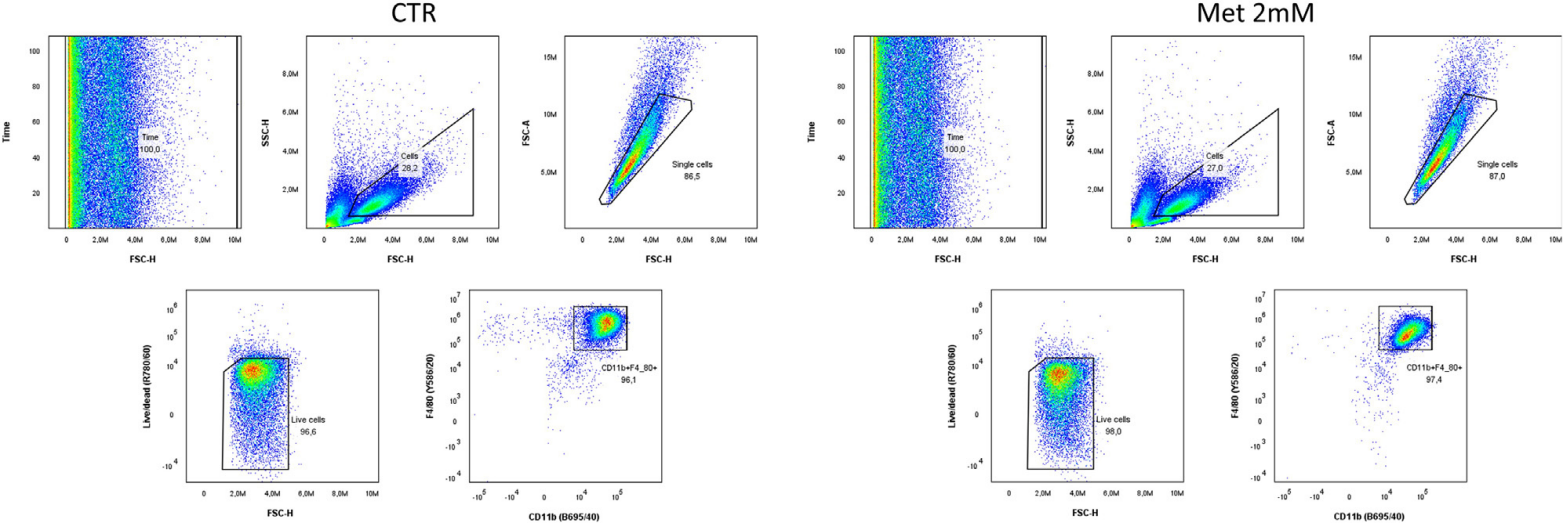
Gating strategy used for the identification of fully differentiated BMDMs The same gating strategy was applied for untreated (CTR) and metformin-treated (Met 2 mM) conditions.

58. Run samples through NovoCyte Quanteon flow cytometer.
59. Assess macrophage differentiation using FlowJo software (see troubleshooting: problem 1).
60. Extract the following data (Figure 3).
  a. Percentage of CD11b^+^ F4/80^+^ cells/live cells (see troubleshooting: problem 2 and problem 3).
  b. Percentage of I-Ab^+^ cells/CD11b^+^ F4/80^+^ cells.
  c. Geometrical mean of I-Ab.
  d. Geometrical mean of H2Kb/H2Db.
  e. Geometrical mean of CD80.
  f. Geometrical mean of CD86.

**Figure 4:**
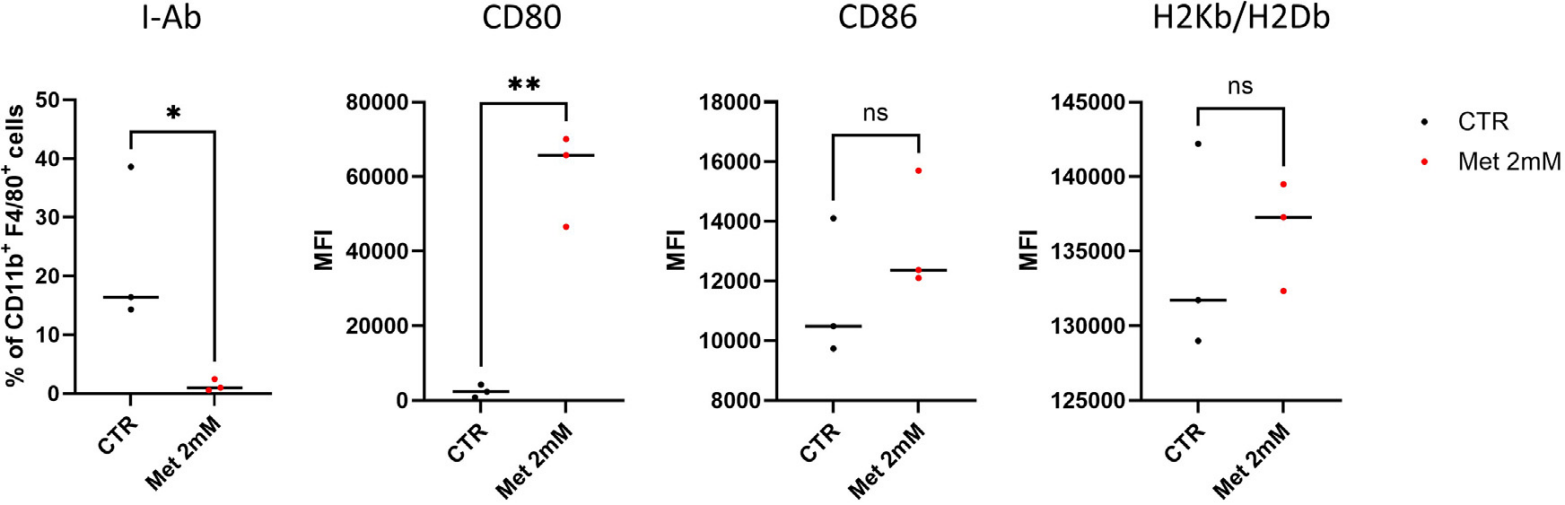
Assessment of specific macrophage markers in metformin-treated and untreated BMDMs Quantification of percentage of I-Ab^+^ cells and expression levels of CD80, CD86, and H2Kb/H2Db shown as mean fluorescence intensity (MFI) in CD11b^+^ F4/80^+^ untreated (CTR) and metformin-treated (Met 2 mM) cells. Unpaired t-test, *n* = 3 biological replicates, ns = p-val>0.05, ∗ = p-val<0.05, ∗∗ = p-val<0.01.

***Note:*** Expression levels of I-Ab, H2Kb/H2Db, CD80, and CD86 can be used to further determine the effects of the specific cues on macrophage functions, such as antigen presentation and T cell activation (Figure 4).

### Actin staining with phalloidin

**Timing: 5 h**

**Figure 5:**
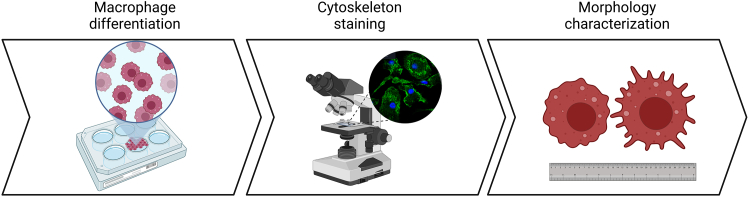
Schematic representation of BMDM cytoskeleton analysis

This protocol section allows the study of morphological changes of BMDMs by immunofluorescence analysis of cytoskeleton actin filaments. After 7 days of differentiation, both untreated and metformin-treated BMDMs are fixed using 4% PFA in order to preserve their cellular shape and assessed for morphology by phalloidin staining (Figure 5).

**CRITICAL:** To carry out immunofluorescence staining to assess morphology or to analyze protein levels of specific macrophage markers, insert sterile glass coverslips in the 6-well plate and seed 5 × 10^6^ cells/well, either with or without treatment, as mentioned in steps 22 to 25.

At the end of differentiation (day 7) on glass coverslips, follow the subsequent steps.

61. Gently remove the cell supernatant.
62. Wash twice with 1× PBS.
63. Incubate BMDMs with 1 mL of 4% PFA for 10 min at 21°C under a chemical hood.
64. Remove the 4% PFA and discard in chemical bin.
65. Wash twice with 1× PBS.
66. For intracellular staining, permeabilize the cells by incubating with PBS supplemented with 0.3% Triton-X-100 for 10 min at 21°C.
67. Wash twice with PBS supplemented with 0.1% Triton-X-100 for 5 min.
68. Wash with 1× PBS for 5 min.
69. Blocking with PBS supplemented with 3% BSA for 30 min at 21°C.
70. Wash 3 times with PBS supplemented with 0.1% Triton-X-100 for 5 min.
71. Dilute phalloidin dye 1:300 in PBS supplemented with 1% BSA to obtain phalloidin working solution.
72. Incubate slides with 500 μL/well of phalloidin working solution at 21°C for 1.5 h covered from light.
73. Wash three times in PBS supplemented with 0.1% Triton-X-100 protected from light for 10 min.
74. While washing, prepare the glass slides by labeling name, date, animal identity, and staining type.

***Note:*** To conduct a blind experiment, we recommend doing the blinding at this stage. Ideally, you will team up with a colleague, who will get the list of animals and treatment of the BMDMs. They will give each animal a different identification number, making sure that the image acquisition is blinded.

75. Lift the coverslips gently out of the wells with a pipette tip and tweezers.
76. Mount the coverslip with Fluoromount medium containing DAPI on a labeled glass slide.
77. Seal the coverslip with nail polish by gently dabbing the tip of the nail polish brush in each corner of the coverslip.
78. Let it dry for 20 min at 21°C before imaging or storing protected from light.

**Pause point:** If you wish, you can store the stained slides at 4°C in a slide box for a week before imaging. However, we recommend imaging the slides the day after the staining.

**Figure 6:**
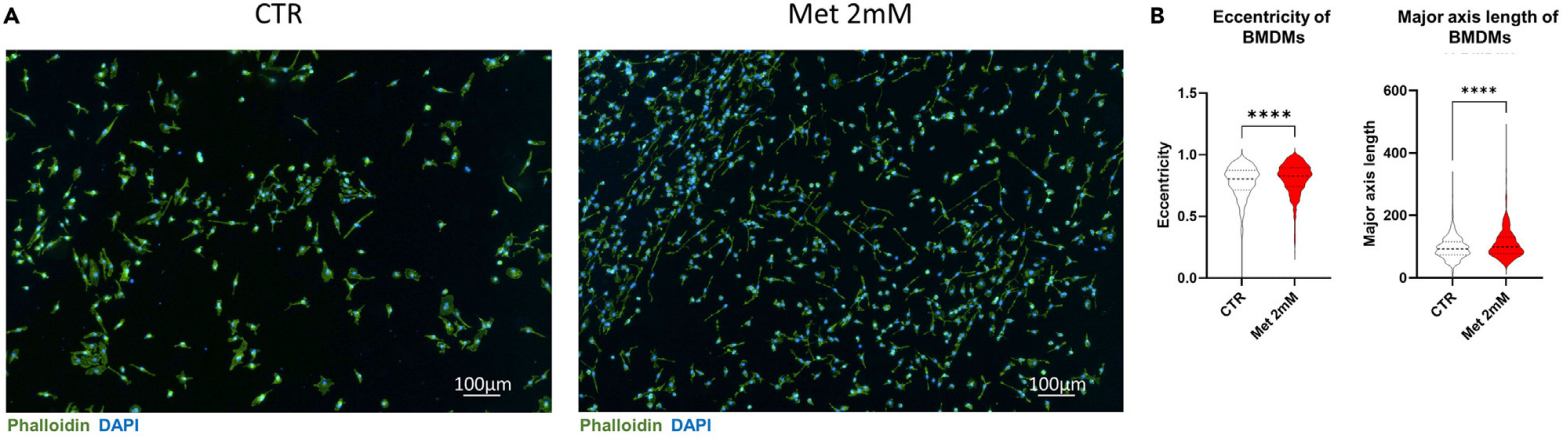
Assessment of cytoskeleton modifications in untreated and metformin-treated BMDMs (A) Untreated (CTR) and metformin-treated (Met 2 mM) BMDMs stained with phalloidin (green) and DAPI (blue). Scale bar: 100 μm. (B) Quantification of eccentricity and major axis length of untreated and 2 mM metformin-treated BMDMs. Dashed lines represent the median while the dotted lines represent the quartiles. Two-tailed Mann-Whitney U test, *n* = 3 biological replicates, more than 300 cells per image in each condition. ∗∗∗∗ = p-val<0.0001.

79. Image cells using Nikon Eclipse confocal microscope (Figure 6).

***Note:*** To quantify morphological features of treated and untreated BMDMs, confocal pictures can be submitted to a software for image analysis, such as ImageJ or CellProfiler. In our original study, we took advantage of CellProfiler.^4^ For further information, refer to CellProfiler website https://cellprofiler.org/.

### Evaluation of macrophage cytotoxicity

**Timing: 3 days**

**Figure 7:**
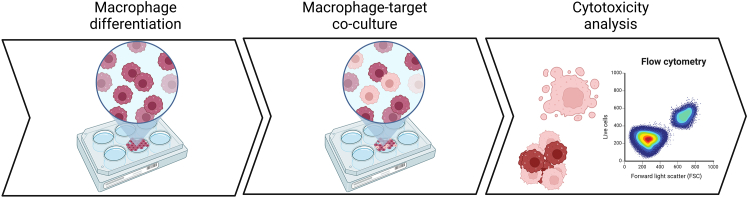
Schematic representation of BMDM co-culture with tumor cells and cytotoxicity analysis

This assay is based on the co-culture of effector cells (i.e., macrophages) with target cells (e.g., murine glioma GL261 cell line, as described in the abovementioned manuscript^1^). The increase in proportion of dead target cells compared to their spontaneous death in culture determines a cytotoxic activity of effector cells. On the other hand, the reduction of target dead cells suggests supportive macrophage functions. The long-lasting CFSE staining allows the discrimination between effector and target cells after co-culture via flow cytometry. Additionally, the utilization of TO-PRO-3 to stain dead cells allows avoiding washes that can cause dead cell loss. To further define the induced death pathway, such as apoptosis, the substitution of TO-PRO-3 with Annexin V and propidium iodide is suggested (Figure 7).

80. After 7 days of differentiation, remove the supernatant from each well of BMDMs.
81. Gently add 2 mL/well of PBS.
82. Remove the supernatant from each well.
83. Add 2 mL/well of complete DMEM.
84. Scrape off the cells using a cell scraper and collect in 15 mL tubes.
85. Fill the 15 mL tubes with 3 mL/tube of complete DMEM.
86. Centrifuge the tube for 7 min at 300 × g at 21°C.
87. Remove the supernatant from each tube of BMDMs.
88. Resuspend the cells in 1 mL of complete DMEM.
89. Count the cells and dilute BMDMs to obtain a solution of 8 × 10^5^ cells/mL.
90. Seed BMDMs (effectors) according to the desired effector/target (E/T) ratio in 96-well plate.
  a. 1/1 E/T ratio: pipette 50 μL/well of cell suspension to seed 4 × 10^4^ cells/well.
  b. 2/1 E/T ratio: pipette 100 μL/well of cell suspension to seed 8 × 10^4^ cells/well
  c. 1/2 E/T ratio: pipette 50 μL/well of cell suspension to seed 4 × 10^4^ cells/well.
  d. Only effector: pipette 100 μL/well of cell suspension to seed 8 × 10^4^ cells/well.

***Note:*** Plan to have for each condition at least three technical replicates. The inclusion of other E/T ratios to define ratio-dependent cytotoxicity is recommended.

91. Fill the wells with complete DMEM to obtain a final volume of 100 μL.
92. Incubate the 96-well plate at 37°C and 5% CO_2_.
93. The day after BMDM seeding, remove the supernatant from the T75 cell culture flask containing GL261 cells.

***Note:*** GL261 cells are cultured in complete DMEM (10% FBS, 1% P/S, 1% HEPES) at 37°C and 5% CO_2_. Since they are adherent cells, they need to be detached using trypsin 0.1% in PBS.

94. Gently add 5 mL/flask of PBS.
95. Remove the supernatant from T75 cell culture flask containing GL261 cells.
96. Add 3 mL/flask of 0.1% trypsin.
97. Incubate 5 min at 37°C and 5% CO_2_.
98. Add 7 mL/flask of complete DMEM.
99. Flush the flask with the supernatant to obtain a single-cell suspension.
100. Collect the cell suspension in 15 mL tube.
101. Centrifuge the tube for 7 min at 300 × g at 21°C.
102. Remove the supernatant from each tube of GL261.
103. Resuspend the cells in 5 mL of complete DMEM.
104. Count the cells and dilute them to obtain a final suspension of 1 × 10^6^ cells/mL.
105. Transfer 2 mL of cell suspension to 5 mL round-bottom polystyrene tubes.

***Note:*** Prepare two tubes for the further steps in order to have both stained and unstained target cells.

106. Fill up the GL261 tubes to 3 mL with PBS.
107. Centrifuge the tubes for 7 min at 300 × g at 21°C.
108. Prepare the stock solution of CellTrace CFSE dye adding 18 μL of DMSO to the CellTrace CFSE dye vial.
109. Dilute 2 μL of CellTrace CFSE stock solution in 1998 μL of PBS supplemented with 0.2% BSA to obtain CellTrace CFSE working solution.
110. Remove the supernatant from each tube of GL261.
111. Add 2 mL of CellTrace CFSE working solution to one tube of GL261 to label them and allow their identification during flow cytometry analysis.
112. Add 2 mL of PBS supplemented with 0.2% BSA to the unlabeled control.
113. Resuspend the cell suspensions via pipetting.
114. Incubate for 10 min at 37°C and 5% CO_2_ the cell suspensions protected from light.
115. Resuspend the cells via pipetting.
116. Incubate for 10 min at 37°C and 5% CO_2_ the cell suspensions protected from light.
117. Add 2 mL of complete DMEM to each tube.
118. Incubate 10 min at 37°C and 5% CO_2_ the cell suspensions protected from light.
119. Centrifuge the tube for 7 min at 300 × g at 21°C.
120. Remove the supernatant from each tube of GL261.
121. Add 4 mL of complete DMEM in each well.
122. Centrifuge the tube for 7 min at 300 × g at 21°C.
123. Remove the supernatant from each tube of GL261.
124. Dilute GL261 to obtain a solution of 8 × 10^5^ cells/mL.
125. Seed CFSE-stained GL261 cells (targets) according to the desired effector/target ratio in 96-well plate.
  a. 1/1 E/T ratio: pipette 50 μL/well of cell suspension to seed 4 × 10^4^ cells/well.
  b. 2/1 E/T ratio: pipette 50 μL/well of cell suspension to seed 4 × 10^4^ cells/well.
  c. 1/2 E/T ratio: pipette 100 μL/well of cell suspension to seed 8 × 10^4^ cells/well.
  d. Only target: pipette 100 μL/well of cell suspension to seed 8 × 10^4^ cells/well.
126. Seed unlabeled GL261 cells at 8 × 10^4^ cells/well in 100 μL.

***Note:*** Seed at least three technical replicates of CFSE-stained and unstained GL261.

**Figure 8:**
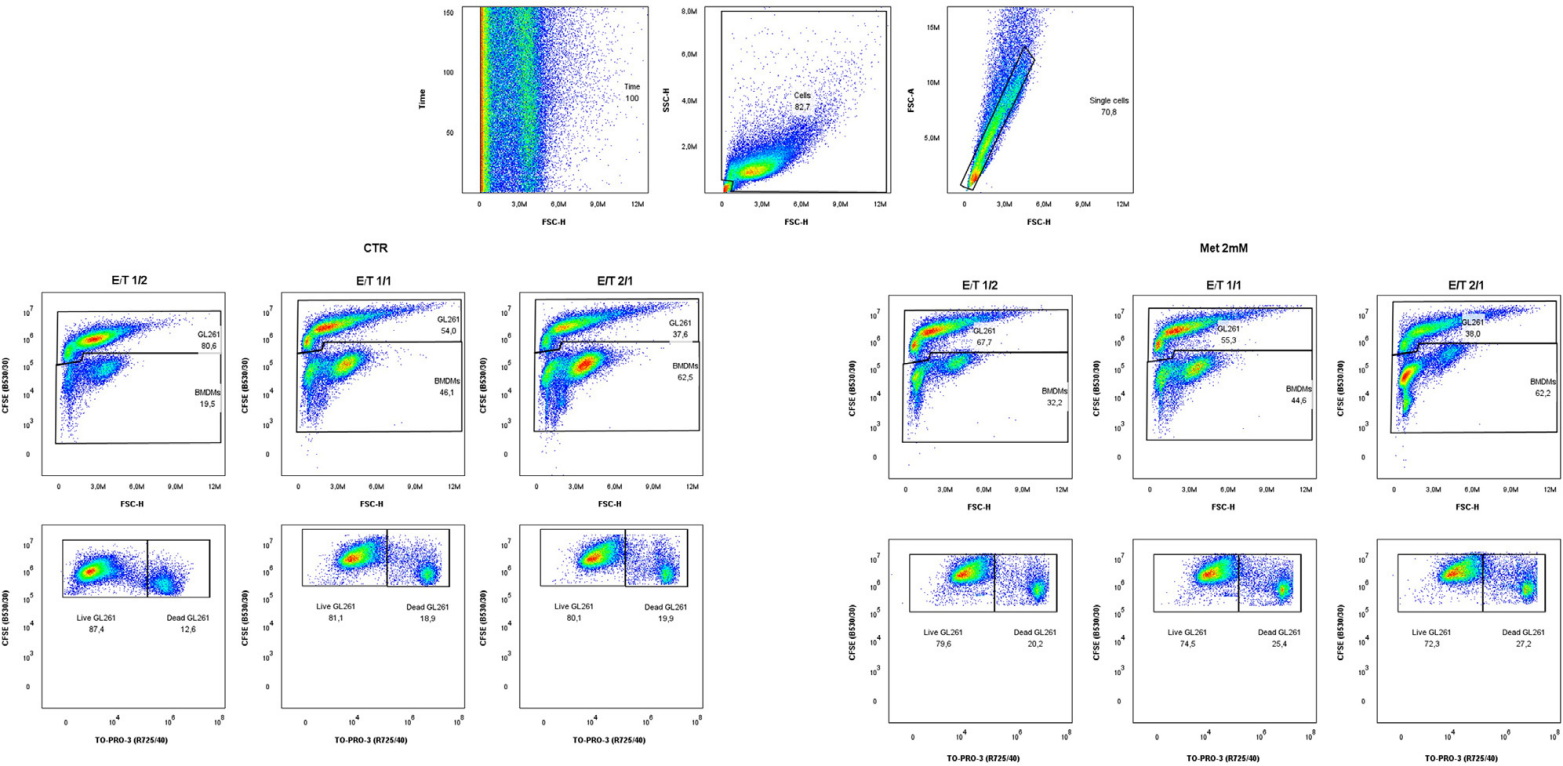
Gating strategy used for the identification of target cells that underwent cytolysis Graphs in the first row show the gates applied to remove debris and doublets in both control (CTR) and metformin-treated (Met 2 mM) BMDMs. Graphs in the second and third raw represent the gating strategy applied for the quantification of target dead cells at different ratios (1/2 left, 1/1 center, 1/1 right) after co-culture with control (CTR) and metformin-treated (Met 2 mM) BMDMs.

127. Add another 100 μL of complete DMEM to obtain a final volume of 200 μL/well.
128. Incubate 96-well plate at 37°C and 5% CO2.
129. The day after the seeding, collect cells by scraping the surface of each well with a pipette tip and transfer to a 5 mL round-bottom polystyrene tube.
130. Add 100 μL of PBS supplemented with 2% BSA to each well to collect all the remaining cells.
131. Add 3 mL of PBS supplemented with 2% BSA to each tube.
132. Centrifuge the tubes for 7 min at 300 × g at 4°C.
133. Dilute 1 μL of TO-PRO-3 in 99 μL of PBS supplemented with 0.2% BSA to obtain TO-PRO-3 working solution.
134. Remove the supernatant from each tube.
135. Resuspend the cells in 100 μL of PBS supplemented with 0.2% BSA.
136. Add 1 μL of TO-PRO-3 working solution to one tube to stain dead cells.
137. Incubate for 10 s at 21°C.
138. Run samples through NovoCyte Quanteon flow cytometer.
139. Repeat steps 67 to 69 for all the tubes.
140. Assess GL261 cell death using FlowJo (Figure 8).
141. Extract the following data from all the samples:
  a. Percentage of BMDMs/cells.
  b. Percentage GL261 cells/total cells (see troubleshooting: problem 4).
  c. Percentage GL261 dead cells/total GL261 cells.

**Figure 9:**
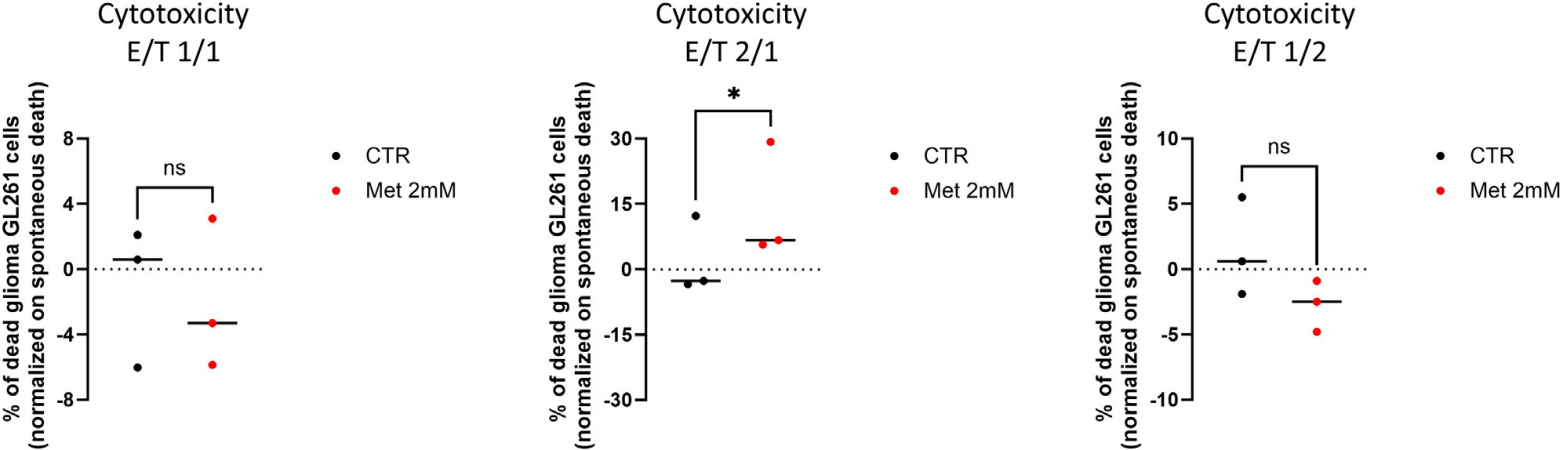
Assessment of BMDM cytotoxicity towards tumor cells Quantification of percentage of dead glioma GL261 cells, normalized on their spontaneous death, after incubation with untreated or 2 mM metformin-treated BMDMs at different E/T ratios. Paired t-test, *n* = 3 biological replicates, ns = p-val>0.05, ∗ = p-val<0.05.

***Note:*** Normalize the percentage of GL261 dead cells/total GL261 cells in co-culture sample with the percentage of dead GL261 cells/total GL261 cells in the samples containing only GL261 cells (spontaneous GL261 cell death) (Figure 9).

### Evaluation of macrophage phagocytic activity with IncuCyte

**Timing: 1–2 days**

**Figure 10:**
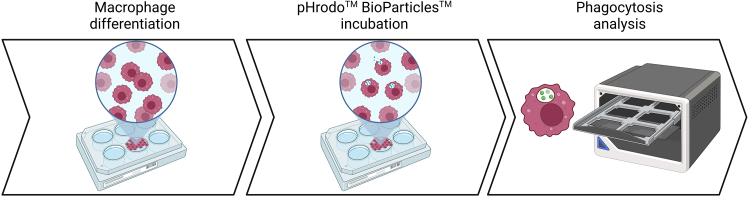
Schematic representation showing the experimental settings used to assess the phagocytic capacity of BMDMs

The usage of pHrodo particles enables an accurate analysis of the phagocytic activity of treated and untreated BMDMs after 7 days of differentiation. The pHrodo particles allow reduction of non-specific signal emitted by particles that have not been engulfed. This is in line with the pH-sensitivity of the pHrodo dye, which does not emit fluorescence in the extracellular space, whereas it produces a signal in the acidic compartments of the cells, such as the lysosomes and endosomes (Figure 10).

***Note:*** When seeding the cells (step 24), consider an additional well per each condition that will be needed for cell counting before the actual start of the phagocytosis assay. Moreover, for each condition, plan at least two wells for technical replicates and one well without pHrodo Red Phagocytosis Particles as negative control.

142. After 7 days of differentiation, remove the supernatant from one well/condition of BMDMs to quantify the amount of differentiated macrophages in each condition.
143. Add 2 mL of PBS to the emptied wells.
144. Remove the supernatant from PBS-containing wells.
145. Add 2 mL of complete DMEM.
146. Collect the cells by scraping the surface of each well with a cells scraper and collect the cell suspension in 15 mL tubes.
147. Repeat steps 145 and 146 to ensure collection of all the cells.
148. Centrifuge the tubes for 7 min at 12000 rpm at 21°C.
149. Remove the supernatant from each tube.
150. Resuspend the cells in 1 mL of complete DMEM.
151. Count the cells.
152. Resuspend one vial of pHrodo Red Phagocytosis Particles in 2 mL of PBS to obtain a stock solution of 1 mg/mL.
153. Vortex for 5 min the vial to completely homogenize the particle suspension.
154. Dilute 10 μL of pHrodo Red Phagocytosis Particles stock solution in 990 μL of complete DMEM to obtain a working solution of 10 μg/mL.
155. Replace the supernatant with fresh complete DMEM.

***Note:*** The compound (e.g. metformin) is needed during the differentiation step, but not for the phagocytosis assay. Some drugs can temporary impact the phagocytic capacity of the cells, so their usage during the assay can mask their effect obtained during the differentiation. The phagocytosis assay is performed without detaching the cells to reduce stress.

156. Add dropwise 100 μL/well of pHrodo Red Phagocytosis Particles working solution.
157. Place the plate in the IncuCyte and incubate for 24 or 48 h.
158. Perform automatic plate scan at 1 h intervals.

***Note:*** Perform first scan before adding pHrodo Red Phagocytosis Particles.

159. Quantify particle engulfment using the IncuCyte 2022A analysis software.

***Note:*** The analysis parameters require adaptation based on the experiment. Adjust size (phase) and threshold (red) values accordingly to determine the area covered by the cells and the engulfed particles, respectively. Activated pro-inflammatory macrophages show thin cellular membranes and light cytoplasm, which reduce the contrast between the plate surface and the cells, complicating the identification of precise cellular borders. To include all the cells in the analysis, adapt the phase confluence parameter to macrophage shape. In case the software recognizes empty pixels as occupied by cells, reduce the “Adjust size” value. Vice versa, if the software does not identify pixels that are occupied by the cells, increase the “Adjust size” value.

**Figure 11:**
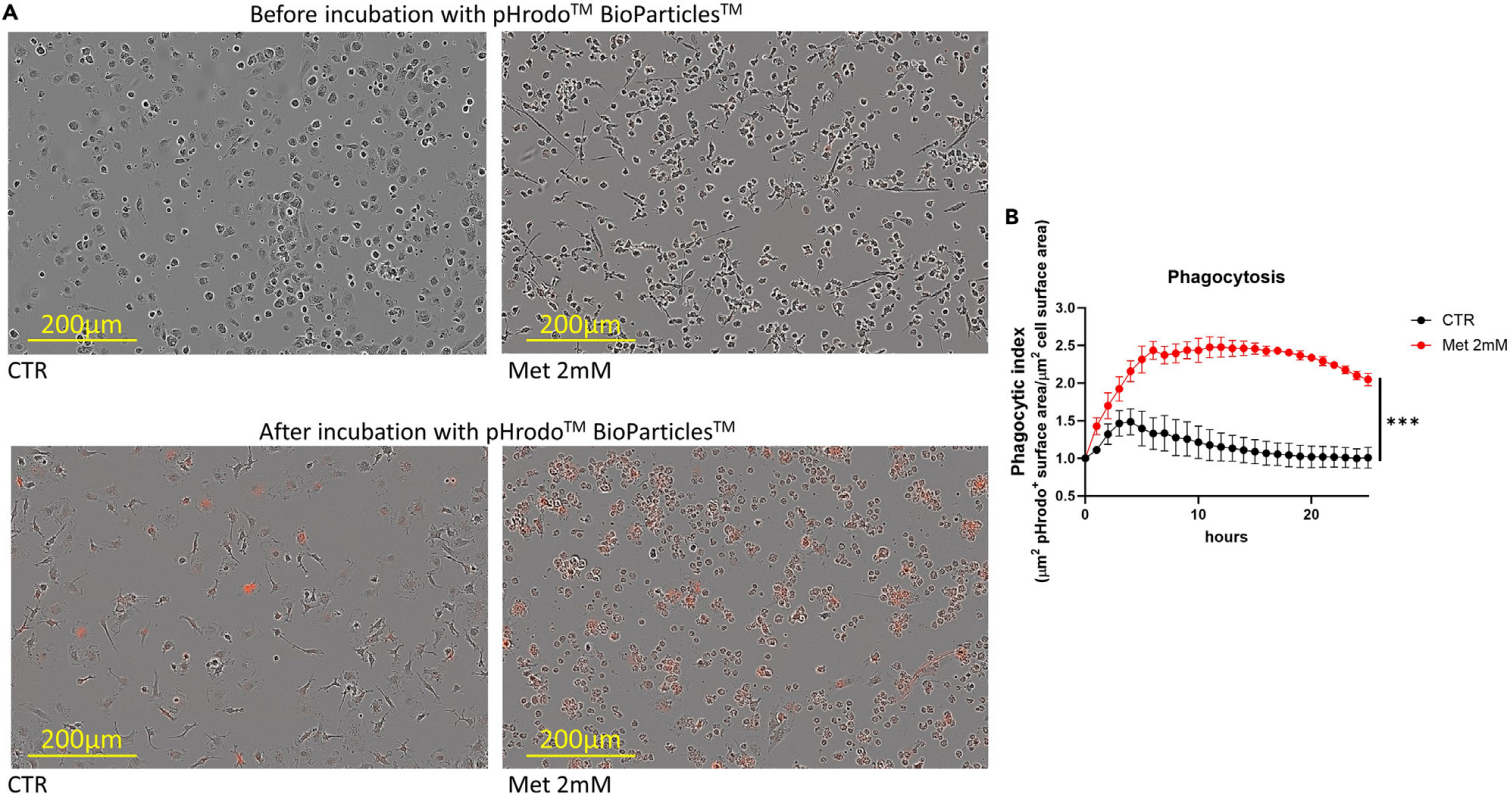
Assessment of BMDM phagocytic capacity (A) Representative IncuCyte images for quantification of engulfed particles. Upper pictures represent the initial state, before incubation with pHrodo bioparticles of untreated (CTR) and 2 mM metformin-treated (Met 2 mM) BMDMs. Lower pictures show untreated (CTR) and 2 mM metformin-treated (Met 2 mM) BMDMs after 25 h incubation with pHrodo bioparticles. Scale bar: 200 μm. Red color represents fluorescence emitted by engulfed pHrodo bioparticles. (B) Quantification of normalized phagocytic index using the IncuCyte analysis software. Graph shows phagocytic index ± SD. 2-way ANOVA, *n* = 3 biological replicates, ∗∗∗ = p-val<0.001.

160. Extract the following values for all the wells at all time-points:
  a. Red area (see troubleshooting: problem 5).
  b. Phase area.
  c. Red area/Phase area (Figure 11).

## Expected outcomes

This protocol provides an established approach to treat bone marrow cells during their differentiation towards macrophages. Additionally, it provides detailed guidelines to address the effects of the treatment on the immunological functions of macrophages.

From each mouse, this protocol leads to 8 to 10 wells of differentiated macrophages.

The protocol leads to an average of 85%–95% live cells at the end of the differentiation. Of these live cells, approximatively 90% of them are CD11b^+^ F4/80^+^ fully differentiated macrophages. Additionally, flow cytometry-based quality control provides information about expression of key functional macrophage markers, such as MHC class I and II molecules and the co-stimulatory molecules CD80 and CD86.

Furthermore, morphological analyses of differentiated macrophages enable the identification of several features, such as compactness and eccentricity.

At the functional level, untreated fully differentiated macrophages do not show cytotoxic activities, while they can promote survival or proliferation of tumor cells. The phagocytic capacity of untreated BMDMs is low, with approximately 20% of the area covered by cells co-localizing with pHrodo fluorescence.

In addition to the abovementioned assays, BMDMs and their supernatants can be harvested to extract proteins, nucleic acids, or metabolites to characterize the effect of the tested cues. Examples of these approaches can be found in Scafidi et al., 2024.^1^

## Quantification and statistical analysis

Based on the specific scientific questions, the data obtained with this protocol can be analyzed applying different statistical tests. In case of comparison between drug-treated BMDMs and untreated control cells for the percentage of differentiated cells or expression of markers, we recommend the usage of an unpaired t-test (single drug at a single dose) or a one-way ANOVA (several drugs and/or at several concentrations). For the comparison of morphological features between different conditions, we recommend using an unpaired t-test (single drug at a single dose) or a one-way ANOVA (several drugs and/or at several concentrations) in which each cell represents a technical replicate for the corresponding biological replicates. For the cytotoxicity assay, in case all the biological replicates are performed with a single-batch of target cells, the optimal approach would be an unpaired t-test (single drug at a single dose) or a one-way ANOVA (several drugs and/or at several concentrations). In case of different batches of target cells, with potentially different levels of spontaneous cell death, an alternative approach would be the paired t-test (single drug at a single dose) or a paired one-way ANOVA (several drugs and/or at several concentrations). Lastly, for the phagocytosis assay, the best approach would be the two-way ANOVA to take into consideration both treatment and time variables.

## Limitations

This protocol is optimized for 6-well plate differentiation of bone marrow cells towards macrophages. Considering the amounts of cells obtained from each mouse, the usage of multiple drugs and/or at different concentrations requires the pooling of cells from different mice for each biological replicate. Direct scale-down from 6-wells to other multi-well plates may not lead to optimal results.

Cytotoxic cues would lead to lower cell confluence when comparing control and treated cells, which may induce alterations of BMDM functions not directly correlated to the drug effect. Similarly, if these cues negatively affect cell differentiation, for example by preventing a complete maturation of the cells towards macrophages, any alteration of the immunological functions may not be a consequence of the treatment. For these reasons, before running functional analyses, carrying out quality control analyses by multicolor flow cytometry is recommended.

## Troubleshooting

### Problem 1

The compensation is not optimal for spectral spillover removal (step 68.c).

### Potential solution

Macrophages and other myeloid cells display high levels of autofluorescence, especially if excited with blue or yellow lasers, coinciding with the peak of emission of the most used fluorochromes FITC and PE. On the other hand, the beads used for compensation do not show autofluorescence. If the compensation made with the beads is not sufficient, perform the single staining on differentiated macrophages. If the problem persists, for example in case a lowly expressed antigen is detected by antibodies labeled with fluorochromes whose emission coincides with macrophage autofluorescence peaks, take into consideration to use different antibodies. Alternatively, the inclusion of FMO controls facilitates the identification of the right positivity threshold, thus overcoming the autofluorescence effect.

### Problem 2

Low cell viability or low percentage of CD11b^+^ F4/80^+^ in all the conditions (step 69.a).

### Potential solution

If bone marrow cells did not differentiate completely or they died overtime in all the conditions, including the untreated control, it may be caused by inappropriate cell culture plate or inefficient activity of the differentiating agent M-CSF. In the first case, make sure that the 6-well plates do not have any coating or enhancer of attachment. Cell scraping should not induce substantial cell death; however, the utilization of dissociating reagents (e.g., trypsin or accutase) can improve the detachment process after optimization. In the second case, verify the storage conditions of M-CSF. It should not undergo several freezing-thawing cycles, so once diluting the stock solution, prepare small aliquots to entirely use them for each experiment and avoid refreezing the leftover.

### Problem 3

Low cell viability or low percentage of CD11b^+^ F4/80^+^ in treated macrophages (step 69.a).

### Potential solution

Specific cues can affect the viability of bone marrow cells and/or differentiated cells. If any abnormal cell death or reduced differentiation ability is detected via multicolor flow cytometry for quality control analyses, perform serial dilutions of the chosen compound and test again for viability and differentiation.

### Problem 4

High level of target cell spontaneous death in cytotoxicity assay (step 181.c.ii).

### Potential solution

If the level of target cell spontaneous death is very high at baseline, it can be due to poor cell culture conditions or the CFSE staining. First, check the viability levels of stained and unstained target cells, because if they are similar, the issue would be in cell culture conditions. To solve this problem, control viability for the last two cell passages in order to have healthy cells for the cytotoxicity assay and avoid any possible pathogen contamination, such as mycoplasma. If the spontaneous death level is considerably higher in stained target cells, perform CFSE dye titration to obtain acceptable fluorescence intensity at lower concentrations. If the problem persists, take into consideration changing the tracking dye.

### Problem 5

Level of phagocytosis is very low in all the conditions (step 207.a).

### Potential solution

The pHrodo Red Phagocytosis Particles tend to clump and form aggregates that normal pipetting cannot dissolve, thus resulting in poor macrophage engulfment. To avoid this phenomenon, vigorously vortex the pHrodo Red Phagocytosis Particles until the solution looks homogeneous. If the problem persists, perform sonication before incubation of the particles with BMDMs.

## Resource availability

### Lead contact

Additional information and requests for resources and reagents should be directed to the lead contact, Alessandro Michelucci (alessandro.michelucci@lih.lu).

### Technical contact

Questions about the technical aspects of performing this protocol should be directed to the technical contact, Andrea Scafidi, andrea.scafidi@lih.lu.

### Materials availability

This study did not generate any new unique reagents.

### Data and code availability

The data included in this study is originally derived from Scafidi et al., 2024.^1^

This study did not generate any code.

## Acknowledgments

We acknowledge financial support from the 10.13039/501100006672Action LIONS Vaincre le Cancer Luxembourg. F.L.-H.M. was supported by the Luxembourg National Research Fund (FNR) through the FNR-PRIDE program i2TRON (PRIDE/14254520/I2TRON). We would like to acknowledge Mr. Alexandros Pailas and Ms. Marta De Lucas Sanz for the introduction to CellProfiler, as well as Dr. Djalil Coowar at the University of Luxembourg Animal Facility and Ms. Anaïs Oudin and Ms. Stephanie Sallai at the Luxembourg Institute of Health Animal Facility for the technical support with the collection of mouse samples. Lastly, we thank Dr. Aurélie Poli for assisting with the setting up of the functional analyses and Ms. Amandine Bernard for helping with the key resources table. For the purpose of open access, and in fulfilment of the obligations arising from the FNR grant agreement, the author has applied a Creative Commons Attribution 4.0 International (CC BY 4.0) license to any Author Accepted Manuscript version arising from this submission. The graphical abstract and the figures were created using BioRender.com.

## Author contributions

A.S. optimized and carried out all animal work, cell culture, flow cytometry, and functional analyses. F.L.-H.M. optimized and carried out actin staining and morphological quantifications. A.M. conceptualized and supervised the project. A.S. and F.L.-H.M. wrote the manuscript draft. All authors reviewed and edited the manuscript.

## Declaration of interests

The authors declare no competing interests.
